# Factors Associated with Stunted Growth in Children Under Five Years in Antananarivo, Madagascar and Bangui, Central African Republic

**DOI:** 10.1007/s10995-021-03201-8

**Published:** 2021-08-12

**Authors:** Pascale Vonaesch, Serge Ghislain Djorie, Kaleb Jephté Estimé Kandou, Maheninasy Rakotondrainipiana, Laura Schaeffer, Prisca Vega Andriatsalama, Ravaka Randriamparany, Bolmbaye Privat Gondje, Synthia Nigatoloum, Sonia Sandrine Vondo, Aurélie Etienne, Annick Robinson, Francis Allen Hunald, Lisette Raharimalala, Tamara Giles-Vernick, Laura Tondeur, Frédérique Randrianirina, Alexandra Bastaraud, Jean-Chrysostome Gody, Philippe Jean Sansonetti, Rindra Vatosoa Randremanana, AFRIBIOTA Investigators, Laurence Barbot-Trystram, Robert Barouki, Alexandra Bastaraud, Jean-Marc Collard, Maria Doria, Aurélie Etienne, Serge Ghislain Djorie, Tamara Giles-Vernick, Bolmbaye Privat Godje, Jean-Chrysostome Gody, Francis Allen Hunald, Nathalie Kapel, Jean-Pierre Lombart, Alexandre Manirakiza, Synthia Nazita Nigatoloum, Lisette Raharimalala, Maheninasy Rakotondrainipiana, Rindra Randremanana, Harifetra Mamy Richard Randriamizao, Frédérique Randrianirina, Annick Robinson, Pierre-Alain Rubbo, Philippe Sansonetti, Laura Schaeffer, Ionela Gouandjika-Vassilache, Pascale Vonaesch, Sonia Sandrine Vondo, Inès Vigan-Womas

**Affiliations:** 1 Unité de Pathogénie Microbienne Moléculaire, Institut Pasteur, 25-28 Rue du Dr Roux, Paris, France; 2 Present Address: Human and Animal Health Unit, Swiss Tropical and Public Health Institute, Socinstrasse 57, 4051 Basel, Switzerland; 3 Present Address: University of Basel, 4051 Basel, Switzerland; 4 Unité D’Epidémiologie, Institut Pasteur de Bangui, Avenue de l’Indépendance, Bangui, Central African Republic; 5 Unité Epidémiologie Et de Recherche Clinique, Institut Pasteur de Madagascar-Ambatofotsikely, BP 1274, 101 Antananarivo, Madagascar; 6 Unité D’Epidémiologie Des Maladies Emergentes, Institut Pasteur, 28 Rue du Dr. Roux, 75015 Paris, France; 7 Centre Pédiatrique de Bangui, Avenue de l’Indépendance, Bangui, Central African Republic; 8 Centre Hospitalier Universitaire Mère Enfant de Tsaralalana, Rue Patrice Lumumba, Rue Mabizo S, 101 Antananarivo, Madagascar; 9 Service de Chirurgie Pédiatrique, Centre Hospitalier Universitaire Joseph Ravoahangy Andrianavalona, Ampefiloha, BP 4150, 101 Antananarivo, Madagascar; 10 Centre de Santé Maternelle Et Infantile de Tsaralalana, Lalana Andriantsilavo, 101 Antananarivo, Madagascar; 11 Anthropology and Ecology of Disease Emergence Unit, Institut Pasteur, 28 Rue du Dr. Roux, 75015 Paris, France; 12 Centre de Biologie Clinique, Institut Pasteur de Madagascar, BP 1274, 101 Antananarivo, Madagascar; 13 Laboratoire D’Hygiène Des Aliments Et de L’Environnement (LHAE), Institut Pasteur de Madagascar, BP 1274, 101 Antananarivo, Madagascar; 14 Present Address: The Center for Microbes, Development and Health, Institut Pasteur of Shanghai and Chinese Academy of Sciences, 411 Hefei Rd, Huangpu, Shanghai, China

**Keywords:** Stunted growth, Risk factors, Central African Republic, Madagascar, Children, Undernutrition

## Abstract

**Objectives:**

With a fourth of all under-five children affected, stunting remains one of the biggest health challenges worldwide. Even though the main underlying factors are known, the exact pathways to stunting varying in affected regions, and interventions thus need to be tailored to the local contexts. This study aimed assessing and comparing factors associated with stunting in two understudied sub-Saharan urban contexts with some of the highest stunting prevalence globally: Bangui, Central African Republic (~ 36%) and Antananarivo, Madagascar (42%).

**Methods:**

We performed a case–control study on 175 + 194 stunted and 237 + 230 non-stunted control children aged 2–5 years and matched for age, gender and district of residency. Factors associated with stunting were identified using a standardized, paper questionnaire delivered by trained interviewers. Statistical analysis was done using logistic regression modelling.

**Results:**

In both sites, formal maternal education lowered the risk of being stunted and restricted access to soap, suffering of anaemia and low birth weight were associated with higher risk of stunting. Short maternal stature, household head different from parents, diarrhoea and coughing were associated with an increased risk and continuing breastfeeding was associated with a lower risk of stunting in Antananarivo. Previous severe undernutrition and dermatitis/ fungal skin infections were associated with higher and changes in diet during pregnancy with lower risk of stunting in Bangui.

**Conclusions:**

Our results suggest maternal education, antenatal care, iron supplementation and simple WASH interventions such as using soap and infection control as general and breastfeeding (Antananarivo) or better nutrition (Bangui) as area-specified interventions.

**Supplementary Information:**

The online version contains supplementary material available at 10.1007/s10995-021-03201-8.

## Significance

Although global trends reveal a decline in stunted child growth, stunting still affects one out of three children in sub-Saharan Africa. Madagascar and the Central African Republic are among the most affected countries globally. Yet we lack data on associated factors in these countries. This article describes nutritional, family, household and socioeconomic factors associated with stunting in the two capital cities, Antananarivo and Bangui. This is the first study specifically assessing stunting-associated factors in these two geographical regions providing a locally adapted roadmap for the implementation of powerful intervention and prevention strategies in these two settings.

## Introduction

Stunted child growth reflects underlying chronic undernutrition and is of major global public health concern. Stunting is characterized by a height of more than two standard deviations below the World Health Organization (WHO) age-specific growth reference standard (Group, 2007). Stunted children have a threefold higher mortality compared to non-stunted children (McDonald et al., 2013; Victora et al., 2021). Children stunted in early life also are at higher risk of displaying cognitive impairments later in life and of experiencing metabolic disease (Prendergast & Humphrey, 2014). Sub-Saharan Africa is one of the most affected regions for child stunting (Local Burden of Disease Child Growth Failure, 2020). The Central African Republic (CAR) and Madagascar, where this study was conducted, are two of the world’s most affected countries, with 42% of Malagasy and 40% of CAR children experiencing stunted growth. This syndrome is associated with several aetiologies, notably a poor, unbalanced diet, insufficient vitamin/micronutrient intake, repeated infections and changes to the gastrointestinal system, especially chronic inflammation and changes to the microbiota (Prendergast & Humphrey, 2014; Vonaesch et al., 2018a). Stunted child growth is also influenced by socio-economic and political factors, including familial resources and configuration, access to health care, and the broader political economic conditions in which children and their families live (Victora et al., 2021). Factors associated with stunting are varying in each context, even if seemingly similar. Indeed, a meta-analysis in 19 countries showed that interventions worked best when country, community and program context were taken into account (Hossain et al., 2017). It is therefore important to know which factors are present in which context in order to tailor interventions to the local need.

Globally, major efforts have been made to decrease stunting prevalence and nutrition-specific and nutrition-sensitive interventions are included in almost all national health plans (Victora et al., 2021). Although the WHO Global Nutrition Targets aim to reduce stunting by 40% by 2025, the CAR and Madagascar have only achieved limited progress in reaching Global Nutrition Targets (Local Burden of Disease Child Growth Failure, 2020).

Because stunting is inherently multifactorial, no single intervention can tackle childhood undernutrition and stunting (Argaw et al., 2019). Rather, this syndrome requires multi-sectorial, coordinated nutrition-sensitive and nutrition-specific interventions that address all conditions facilitating chronic childhood undernutrition (Heidkamp et al., 2021). As associated factors are context-specific, local studies providing in-depth understanding of factors contributing to stunting are crucial to identify successful intervention strategies for a given context (Carter et al., 2020).

The objective of this study was to assess and compare factors associated with stunted child growth in two sub-Saharan African cities having among the highest prevalence of stunted child growth worldwide: Bangui, the capital of the CAR and Antananarivo, the capital of Madagascar.

## Methods

### Study Design and Study Population

**Figure 1:**
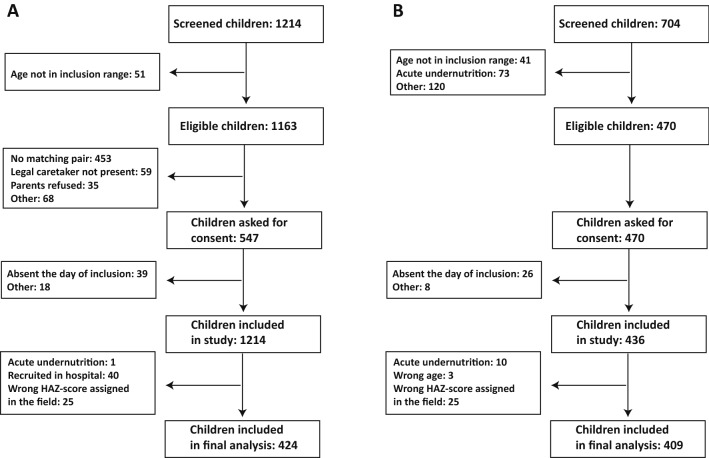
Flow-chart of the children included in the final analysis. Data is summarized for children included in **A** Antananarivo, Madagascar and **B** in Bangui, Central African Republic

The AFRIBIOTA study (Vonaesch et al., 2018b) is a case–control study for stunting in children aged 2–5 years in Bangui, CAR and Antananarivo, Madagascar. The choice of the two study sites was based on the high stunting prevalence and the fact that the consortium worked previously together on a project on diarrhoeal diseases (Breurec et al., 2016; Randremanana et al., 2016). Inclusion criteria were HIV-negative children, neither suffering from acute malnutrition nor from any other severe disease, living in the 6st, 7st or 8th district of Bangui or in two neighbourhoods of Antananarivo (Ankasina or Andranomanalina Isotry). Included children were admitted to the hospital for sample collection and anthropometric measurement. Assuming an alpha-error of 0.05%, a power of 80% and an expected 20% exposure in the cases, we needed at least 169 children per group, hence 676 individuals in the two countries. The final sample size analysed in this study was of 836 children (Fig. 1). Stunted and control children were matched according to age in years, gender, neighbourhood and season of inclusion (dry or wet season). Recruitment took place between December 2016 and March 2018 in Antananarivo and between January 2017 and May 2018 in Bangui. Detailed recruitment procedures are given in the Supplementary methods and the study protocol (Vonaesch et al., 2018b).

### Anthropometric Measurements

Height was measured by trained personnel to the nearest 0.1 cm in a standing position using collapsible height boards (Antananarivo: ShorrBoard Measuring Board, Maryland, USA; Bangui: height board provided by UNICEF); weight was measured to the nearest 100 g using a weighing scale (Antananarivo: KERN, ref. MGB 150K100 and EKS, Inter-équipement Madagascar; Bangui: weighting scale provided by UNICEF) and mid-upper arm circumference (MUAC) was measured using commercial MUAC tape (provided by UNICEF) to the nearest 0.1 cm. Cut-offs were based on the official cut-offs defined by WHO (Onis, 2006).

### Data Collection

A standardized, paper questionnaire in French that was translated ad hoc to the local languages Sangho and Malagasy was used in both study sites and data was entered in double in an Access database. The questionnaire included information about children’s age, gender, family structure, socioeconomic status indicators, sanitary indicators, data about the mother’s pregnancy and child’s and family nutrition and feeding practices. A wealth index was created based on the minimal set of assets, leading to a separation of subjects in three distinct groups in a principal component analysis (PCoA). Details of the wealth index are given in the extended methods. Each child was further examined for comorbidities and venous blood was collected. Complete blood count, C-reactive protein (CRP) and ferritin levels were measured. Ferritin levels were corrected for systemic inflammation (Thurnham et al., 2010), haemoglobin values were adjusted for altitude (Centers for Disease, 1989; Sullivan et al., 2008) and anaemia was defined as less than 110 g/l, according to WHO criteria (OMS, 2011; Onis, 2006). A dietary diversity score (DDS) was calculated based on a 24 h recall (World Health Organization, 2007a, 2007b). Mother’s nutritional status was based on the Body Mass Index (BMI). Non-pregnant mothers were classified by BMI categories as defined by the WHO and pregnant mothers according to the categories proposed by Ververs (Ververs et al., 2013). Clinical parameters such as cough (observed and reported by the mothers), dermatitis (as visible dermal affections of various origins diagnosed by a medical doctor), diarrhoea (> 3 loose stools/day), and tooth decay were assessed during a clinical examination. Previous episode of severe acute malnutrition and perceived low birth weight was based on mother’s recall.

### Statistical Analysis

The statistical analysis was performed with Stata 13. Significance level was fixed for all analyses at 0.05 and all tests were performed bilaterally. Categorical variables were expressed as percentages; quantitative variables were expressed as a mean (± Standard Deviation) or median (interquartile range). The stunted vs. non-stunted groups were compared using Chi^2^ or Fisher Exact test for qualitative variables and the Student *t* test or the Mann–Whitney U test for quantitative variables. All variables were assessed in a bivariate analysis. Factors associated with stunting in bivariate analysis with a P value of < 0.2 were checked for potential confounding factors and interactions and then included in a backward logistic regression. As we did not get a perfect matching for age, gender and season of inclusion, these variables were forced in the multivariate model. Results are reported as adjusted OR with 95% CI, corrected for age in years, gender, season of inclusion and country of origin.

## Results

### General Characteristics of Study Participants and Their Mothers

The final dataset analysed comprised 409 children from Bangui and 424 children from Antananarivo (Fig. 1; Table 1). The general characteristics of the study population are summarized in Table 1. In both study sites, most children lived with their mothers (Bangui: 93.0%, Antananarivo: 97.0%, P = 0.01) and with their fathers (Bangui: 75.0%; Antananarivo: 82.0%, P = 0.02). Bangui households tended to have a higher socioeconomic score (11.0% in lowest, 81.9% in middle and 7.1% in highest income group) compared to Madagascar (66.0% in lowest, 12.2% in middle and 3.8% in highest income group, P < 0.001). In both countries, most families had either the father or the mother working. Nevertheless, 10.6% of Antananarivo children and 22.5% of Bangui children lived in a household in which someone other than the father or mother was the primary earner (P < 0.001). Mothers’ characteristics are summarized in Table S1.

**Table 1:** General description of study population

	Bangui N = 409	Antananarivo N = 424	P value
Gender			Pr = 0.18
Female	205 (50%)	232 (55%)	
Male	204 (50%)	192 (45%)	
Height-for-age z-score			Pr = 0.68
Not stunted (WHZ ≥ − 2)	237 (57%)	230 (54%)	
Moderately stunted (WHZ ≥ − 3 < − 2)	87 (21%)	95 (22.5%)	
Severely stunted (WHZ < − 3)	88 (22%)	99 (23.5%)	
Age			Pr < 0.001
2–3 years	167 (41%)	131 (31%)	
3–4 years	141 (34.5%)	135 (32%)	
4–5 years	101 (24.5%)	158 (37%)	
Socioeconomic score			Pr < 0.001
Lowest score	45 (11%)	280 (66%)	
Middle score	335 (82%)	128 (30%)	
Highest score	29 (7%)	16 (4%)	
Mother and/or father are working			Pr = 0.78
Yes	388 (95%)	404 (95%)	
No	21 (5%)	20 (5%)	
Lives with extended family (uncles or cousins)			Pr < 0.001
Yes	265 (65%)	80 (19%)	
No	144 (35%)	344 (81%)	
Persons living in the household	8.5 (8.1; 8.9)	4.8 (4.6; 5.0)	Pr < 0.001
Children below 5 years living in the household	2.2 (2.1; 2.3)	1.4 (1.4; 1.5)	Pr < 0.001
Animals in household	145 (35%)	153 (36%)	Pr = 0.85

### Nutritional Habits and Young Child Feeding

Roughly a quarter of mothers in Bangui and 5.0% in Madagascar changed their nutrition during pregnancy. The most commonly reported changes were more fruits, vegetables, meat and fish in Antananarivo, and increased consumption of dishes like “Dadawa” (fermented beans that season dishes and are understood to be highly nutritious in central and west Africa), “Kôkô” (*Gnetum* species, which grow in forest tangle and serve as the basis for leaf sauces), “Goussa” (squash seeds, pounded and used in sauces), “Nguiriki” (sauce) and other dishes rich in beans, peanuts, palm oil, meat, fish, caterpillars and green leafy vegetables in Bangui. Mothers claimed that these foods were “highly nutritious”.

**Figure 2:**
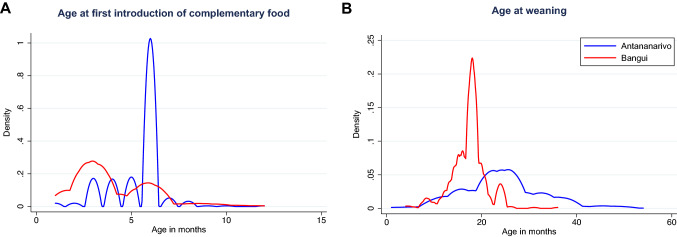
Breastfeeding characteristics in the two study sites. Kernel density plots of **A** the age of first introduction of complementary food and **B** the age of weaning as reported by the mother/primary caregiver. Data from Antananarivo, Madagascar is indicated in blue, data from the Bangui, Central African Republic (CAR) in red

Mothers or caregivers introduced complementary food to children earlier [median of 3 (IQR 1–10) vs. 6 (IQR: 1–8) months; Pr < 0.0001] and breastfed for a shorter period [17.3 (IQR: 16–19) vs. 24.6 (IQR: 16–19) months on average; Pr < 0.0001] in Bangui than in Antananarivo. (Fig. 2, Table 2). Eighty percent (80.0%) of all Bangui children and 90.0% of Antananarivo children were at least partially breastfed throughout their first year of life.

**Table 2:** Description of early life and current nutrition

	Bangui N = 409	Antananarivo N = 424	P value
Nutrition changed during pregnancy			Pr < 0.0001
Yes	99 (24%)	23 (5.5%)	
No	278 (68%)	395 (93%)	
Does not remember/no information	32 (8%)	6 (1.5%)	
Perceived birth weight*			Pr < 0.0001
Smaller than average	36 (9%)	103 (24%)	
Average	188 (46%)	146 (34.5%)	
Bigger than average	50 (12%)	171 (40.5%)	
Not known	135 (33%)	4 (1%)	
Introduction to complementary foods (months)	4.0 (3.8; 4.2)	5.4 (5.3; 5.5)	Pr < 0.0001
Exclusive breastfeeding for 6 months			Pr < 0.0001
Yes	115 (28%)	280 (66%)	
No	289 (71%)	140 (33%)	
Unknown	5 (1%)	4 (1%)	
Complete weaning (months)	17.3 (16.9; 17.7)	24.4 (23.5; 25.2)	Pr < 0.0001
Caregiver changes food items if child seems undernourished			Pr < 0.0001
Yes	184 (45%)	66 (15.5%)	
No	222 (54%)	357 (84.25%)	
Unknown	3 (1%)	1 (0.25%)	
Drinking water source			Pr < 0.0001
Only tap water or water from fountain/bottled water	52 (13%)	421 (99%)	
Other (rain/well water)	357 (87%)	3 (1%)	
Food diversity score	3.6 (3.5; 3.7)	3.9 (3.8; 4.0)	Pr = 0.0012
Snacks during the day	1.1 (1.0; 1.2)	2.6 (2.4; 2.8)	Pr < 0.0001

*As perceived and remembered by the main caregivers

In Antananarivo, the most common complementary food items reported by caregivers was “Koba aina” (enriched ready-to-cook product consisting of corn, rice and peanuts), combined in some households with certain vegetables (potatoes, carrot) or commercially available wheat- and milk-based children’s powder (“Farilac”), milk, biscuits or cheese. Only 11/424 (2.5%) of caregivers reported having regularly given their infant meat and 2/424 (0.5%) fish during the weaning period. In Bangui, the majority of caregivers gave their children a maize and water-based runny porridge (“Koulou”) and less frequently a rice porridge, commercial wheat- and milk-based children’s powder (“phosphatine”, “Bledina”) or very rarely a plantain- or millet-based porridge. In total, 38/409 (9.0%) reported combining this base on a routine basis with vegetables (green leafy vegetables and/or tomatoes), 19/409 (4.5%) with fish, 10/409 (2.5%) with meat, 7/409 (1.5%) with peanuts, 2/409 (0.5%) with eggs, 2/409 (0.5%) with legumes and 1/409 (0.3%) with caterpillars. In both countries, nutritional diversity was on average below the recommended food diversity score of 4 (Table 2) yet 64% of families in Antananarivo and 54% Bangui reporting having given their children at least four different food groups the previous day. Fifty three percent (53%) of families in Antananarivo and 41% of families in Bangui reported having provided their children with meat, and 24% and 45%, respectively, offered fish in the previous day.

Based on anthropological observations in the communities and in accordance with other studies performed in Madagascar (Rakotomanana et al., 2017a, 2017b; Rakotonirainy et al., 2018) we suspect meat and fish quantities to be minimal, and diet in both countries to be primarily starch-based.

### Sanitary Conditions

Sanitary conditions in both settings were challenging for families and are summarized in Table S2. In Bangui, only 16.0% of the households had constant access to soap, compared to 45.0% in Antananarivo. Thirty four percent (34%) of all households in Bangui and 56% in Antananarivo reported to be exposed to a possible contamination source (open toilet, trash bin, wastewater evacuation canal) with a distance of less than 5 m to their house. In 5% of Antananarivo households and 21% of all Bangui households, poultry lived with the family in the same dwelling.

### Prevalence of Micronutrient Deficiencies and Other Co-morbidities

In both countries, children displayed several comorbidities (Table S3). Anaemia was very widespread, with 47% of Bangui children and 20% of Antananarivo children showing haemoglobin levels below 11 g/l. Urinary iodine levels were only measured in Antananarivo. The median level was 87 ug/l (IQR 35; 170), showing moderate iodine deficiency compared to WHO references values for school age children (World Health Organization, 2007a, 2007b). Other observed comorbidities on the day of inclusion included mild respiratory infections, visible caries (38% in Antananarivo), dermatitis and diarrhoea. Virtually all Bangui children (96%) and roughly a quarter (28%) of Antananarivo children did not have full vaccine coverage.

### Factors Associated with Stunted Growth

In Antananarivo, social factors such as mother’s height, mother’s education and a head of household other than the parents were significantly associated with stunted child growth (Table 3). Comorbidities at the time of inclusion, including cough (aOR1.73; CI 1.04; 2.88), diarrhoea (aOR 15.15; CI 1.48; 154.58) and anaemia (aOR 3.60; CI 1.88; 6.88) were significantly associated with stunted child growth, as were early life factors such as perceived birth weight (aOR 2.23; CI 1.20; 4.16 of low vs. normal and aOR 0.30; CI 0.17; 0.54 of high vs. normal) or prolonged breastfeeding (aOR 0.30, CI 0.13; 0.70 of at least one year breastfeeding vs. less). Not having constant access to soap was associated with an 80% increase in stunted child growth.

**Table 3:** Factors associated with stunting, Antananarivo, Madagascar, 2016–2018*

	Non-stunted N = 222 (%)	Stunted N = 184 (%)	Unadjusted OR (95% CI)	Adjusted OR (95% CI)^a^
Socioeconomic score				
Lowest	136 (61)	131 (71)	1.84 (1.82; 2.85)	1.55 (0.90; 2.67)
Middle	82 (37)	43 (23)	Ref	Ref
Highest	4 (2)	10 (5)	4.77 (1.41; 16.10)	9.70 (2.14; 43.93)
Mothers education				
None	4 (2)	9 (5)	Ref	Ref
Primary	94 (42)	101 (55)	0.48 (0.14; 1.60)	0.39 (0.10; 1.50)
Middle school	107 (48)	68 (37)	0.28 (0.08; 0.95)	0.25 (0.06; 0.97)
High school or more	17 (8)	5 (3)	0.13 (0.03; 0.61)	0.06 (0.01; 0.37)
Mother’s height				
More than 150 cm	61 (27.5)	84 (45.5)	Ref	Ref
Less than 150 cm	161 (72.5)	100 (54.5)	2.22 (1.47; 3.35)	2.11 (1.28; 3.47)
Unknown	0 (86)	0 (86)	–	–
Main earner is different from parents				
No	210 (94.5)	164 (89)	Ref	Ref
Yes	12 (5.5)	20 (11)	2.13 (1.01; 4.49)	3.15 (1.25; 7.91)
Partially breastfed ≥ 12 months				
No	14 (6.5)	24 (13)	Ref	Ref
Yes	189 (85)	141 (76.5)	0.44 (0.22; 0.87)	0.30 (0.13; 0.70)
Unknown	19 (8.5)	19 (10.5)	0.58 (0.23; 1.46)	0.41 (0.13; 1.29)
Soap available in household				
Yes	133 (60)	80 (43.5)	Ref	Ref
No or only sometimes	89 (40)	104 (56.5)	1.94 (1.31; 2.89)	1.78 (1.09; 2.90)
Anemia (Hb < 11 g/l)				
No	192 (86.5)	126 (68.5)	Ref	Ref
Yes	26 (11.5)	51 (27.5)	2.99 (1.77; 5.04)	3.60 (1.88; 6.88)
Unknown	4 (2)	7 (4)	2.67 (0.76; 9.30)	3.31 (0.74; 14.74)
Birth weight**				
Average	70 (31.5)	68 (37)	Ref	Ref
Smaller than average	33 (15)	67 (36.5)	2.09 (1.23; 3.56)	2.23 (1.20; 4.16)
Bigger than average	118 (53)	48 (26)	0.42 (0.26; 0.67)	0.30 (0.17; 0.54)
Unknown	1 (0.5)	1 (0.5)	1.03 (0.06; 16.79)	1.01 (0.003; 281.38)
Diarrhea at time of inclusion				
No	221 (99.5)	174 (94.5)	Ref	Ref
Yes	1 (0.5)	10 (5.5)	12.7 (1.61; 100.17)	15.15 (1.48; 154.58)
Coughing at time of inclusion				
No	152 (68.5)	104 (56.5)	Ref	Ref
Yes	70 (31.5)	80 (43.5)	1.67 (1.12; 2.51)	1.85 (1.11; 3.07)
Chicken in household				
No	214 (93)	169 (87)	Ref	Ref
Yes	16 (7)	25 (13)	1.98 (1.02; 3.82)	2.27 (1.04; 4.97)

*Analysis performed on 409 subjects

**As perceived and recalled by the main caregiver

^a^Adjusted for season of inclusion, age in years, gender and country of inclusion (matching) as well as the other independently associated variables

In Bangui, the main factors associated with stunted child growth were maternal education (85% fewer stunted children born to mothers who completed high school compared to no formal education) and different biological factors, such as anaemia (aOR 2.2; CI 1.27; 3.82), low birth weight (aOR 2.81; CI 1.13; 6.98 for small vs. normal weight children), previous episode of severe acute undernutrition (aOR 5.52; CI 1.9; 16.07) and dermatitis at time of inclusion (aOR 4.32; CI 2.08; 8.99). Not having constant access to soap was associated with more than threefold increase in stunted child growth (aOR 3.38; CI 1.68; 6.80). The results of the multivariate analysis of factors associated with stunting in Bangui are summarized in Table 4 and from the two countries pooled in Table S4.

**Table 4:** Factors associated with stunting, Bangui, Central African Republic, 2017–2018*

	Non-stunted N = 223 (%)	Stunted N = 152 (%)	Unadjusted OR (95% CI)	Adjusted OR (95% CI)^a^
Mothers education				
None	8 (3.5)	8 (5)	Ref	Ref
Primary	80 (36)	63 (41.5)	0.79 (0.28; 2.21)	0.94 (0.29; 3.06)
Middle school	87 (39)	74 (48.5)	0.85 (0.30; 2.38)	1.04 (0.32; 3.36)
High school or more	47 (21)	6 (4)	0.13 (0.03; 0.47)	0.17 (0.04; 0.71)
Unknown	1 (0.5)	1 (0.5)	1 (0.05; 18.91)	1.98 (0.06; 65.12)
Soap available in household				
Yes	206 (92)	109 (72)	Ref	Ref
No or only sometimes	17 (8)	43 (28)	4.78 (2.60; 8.78)	3.38 (1.68; 6.80)
Nutrition changed during pregnancy				
No	153 (68.5)	127 (83.5)	Ref	Ref
Yes	70 (31.5)	25 (16.5)	0.43 (0.26; 0.72)	0.36 (0.20; 0.67)
Anemia (Hb < 11 g/l)				
No	127 (57)	51 (33.5)	Ref	Ref
Yes	70 (31.5)	77 (50.5)	2.74 (1.73; 4.33)	2.20 (1.27; 3.82)
Unknown	26 (11.5)	24 (16)	2.30 (1.21; 4.37)	2.36 (1.07; 5.17)
Birth weight**				
Average	101 (5.5)	21 (14)	Ref	Ref
Smaller than average	12 (45)	71 (47)	2.49 (1.15; 5.38)	2.81 (1.13; 6.98)
Bigger than average	30 (13.5)	20 (13)	0.95 (0.50; 1.80)	0.76 (0.35; 1.65)
Unknown	80 (36)	40 (26)	0.71 (0.44; 1.16)	0.73 (0.40; 1.33)
Dermatitis at time of inclusion				
No	205 (92)	117 (77)	Ref	Ref
Yes	18 (8)	35 (23)	3.41 (1.85; 6.28)	4.32 (2.08; 8.99)
Previous episode of severe acute undernutrition				
No	217 (97)	126 (83)	Ref	Ref
Yes	6 (3)	26 (17)	1.64 (1.29; 2.08)	5.52 (1.90; 16.07)

*Performed on 375 subjects

**As perceived and recalled by the mother

^a^Adjusted for season of inclusion, age in years, gender and country of inclusion (matching) as well as the other independently associated variables

## Discussion

Even though Madagascar and the CAR are among the most affected countries globally, we have little data related to factors associated with stunting in these two settings. Our study contributes therefore important data on stunting-associated factors, which can serve as baseline for targeted interventions by policy makers.

In our study low birth weight, lack of access to soap as well as comorbidities such as anaemia, diarrhoea, dermatitis or respiratory disease are risk factors and higher education of the mother is a protective factor for stunting in both study settings, reflecting the most common stunting-associated factors globally (Hawkes et al., 2020).

Roughly a fifth of Antananarivo children and half of Bangui children included had haemoglobin levels indicative of anaemia. We observed similar levels of low iron in Antananarivo (21%), but only a small proportion of children in Bangui had low iron levels (9%). This discrepancy between iron levels and anaemia warrants further investigation. Our urinary iodine measurements in Antananarivo align with a previous report, which showed moderate iodine deficiency across the country. Madagascar is currently reviving a salt-based iodine supplementation program (Randremanana et al., 2019). We did not find any association between food diversity and stunting in the two study sites. These observations clearly necessitate more thorough mixed-methods studies that assess consumed food items and their quantification.

In a study in 137 low- and middle-income countries (LMIC), poor sanitation has been identified to be worldwide the second largest burden to child stunting after low birth weight (Danaei et al., 2016). In contrast to this observation are three recent, very well designed and large-scale randomized clinical trials assessing the benefit of water, sanitation and hygiene (WASH) interventions on child stunting. None of these trials found an effect of WASH on stunting (Cumming et al., 2019; Humphrey et al., 2019; Prendergast et al., 2019). Researchers of these studies acknowledge that interventions must target local exposures, enteric disease burden and cofactors, an approach they termed “transformative WASH”. In accordance, in a recent systematic review assessing fourteen programs to reduce stunting in 19 LMIC, the authors found that similar interventions led to different outcomes in different contexts (Hossain et al., 2017). In our study, of all of the sanitation variables tested, only the presence of soap protected against stunting. This suggests that hand hygiene might be the easiest and most effective WASH intervention to be implemented in Antananarivo and Bangui.

Animals, especially if reared in the same physical space as children, have previously been reported to be associated with stunted child growth (Monira et al., 2020); although these associations vary across different settings (Headey et al., 2017). In our study, roughly a third of all Antananarivo households and more than two-thirds of Bangui households owned poultry. In Antananarivo, owning poultry was associated with a roughly two-fold higher risk of stunting in the multivariate model. No association between poultry and stunting was observed in Bangui. This discrepancy between the two study sites could be due to the fact that Antananarivo is much more densely populated than Bangui, leaving animals in closer proximity to their owners and their children. Indeed, faecal material from chicken and pigs can potentially lead to zoonotic diseases, such as salmonellosis, cryptosporidiosis, pathogenic *E. coli infections*, giardiasis and *Campylobacter* infections, all of which have been associated with stunted child growth (Rogawski et al., 2017; Sanchez et al., 2020). Our data assessing the microbiome of children in the same setting shows that faecal asymptomatic carriage of pathobionts (especially the genera *Shigella/Escherichia* and *Campylobacter*) is associated with stunted child growth (Vonaesch et al., 2018a). Targeted studies on asymptomatic pathogen carriage and their relation with child stunting are currently ongoing.

**Fig. 3:**
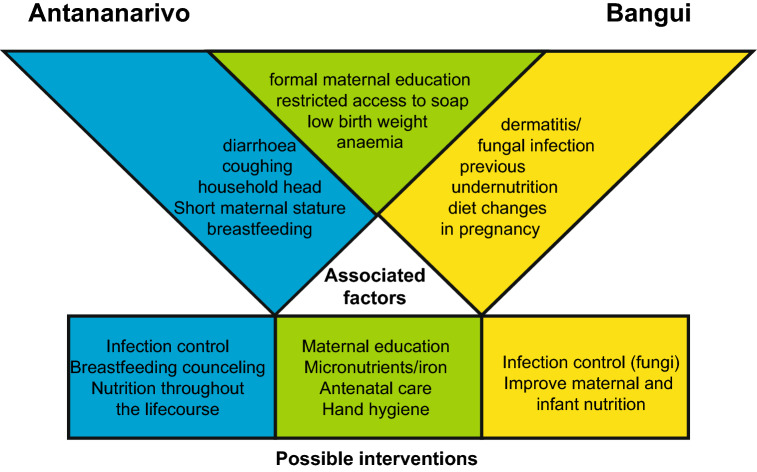
Shared and context-specific factors associated with stunting and suggested interventions. Shared factors and interventions are shown in green, factors and interventions specific to Antananarivo in blue and specific to Bangui in yellow

Beside shared factors, the two study sites also displayed specific factors associated with stunting (Fig. 3).

In Antananarivo, short maternal stature, household head different from parents, diarrhoea and coughing were associated with an increased risk and continuing breastfeeding was associated with a lower risk of stunting. This suggests that in Antananarivo, beside anaemia and low birth weight, infections are one of the main pathways leading to stunted child growth.

Further, the highest socio-economic group seemed to have the highest odds of being stunted. A possible explanation to this puzzling result might be that the recruitment was taking place in two of the poorest neighborhoods in Antananarivo, hence stratifying the poor population to three categories (the highest category representing the richest amongst the poor) rather than assessing for families, which indeed do have a higher socioeconomic score. It is further possible that this socioeconomic group has specific behavioral patterns that are favoring stunting that have not been captured through our data collection. Anthropological studies are ongoing in both study sites.

Previous studies outside the capital city found child gender and age (McCuskee et al., 2018; Rakotomanana et al., 2017a, 2017b; Remonja et al., 2017), low maternal stature (McCuskee et al., 2018; Rakotomanana et al., 2017a, 2017b) and birth weight (McCuskee et al., 2018; Remonja et al., 2017), inadequately spaced birth (Remonja et al., 2017), low paternal height (McCuskee et al., 2018), socioeconomic score (Remonja et al., 2017), infection with the helminth *Trichuris trichiura* (Remonja et al., 2017) and food diversity score (Aiga et al., 2019), to be associated with stunted child growth. This suggests that the pathways leading to stunting in the capital city are slightly different than in other parts of the country, calling for context-specific interventions.

In Bangui, previous severe undernutrition and dermatitis/fungal skin infections were associated with higher and changes in diet during pregnancy with lower risk of stunting. Together with the very high prevalence of anaemia and the low levels of breastfeeding, stunted child growth in Bangui may be primarily related to nutritional deficits, both, in pregnant mothers as well as their infants, thus suggesting the implementation of nutrition-specific interventions.

The main strength of our study lies in the extensive cohort with almost 1000 children and its rigorous control using standardized questionnaires, enabling robust statistical analysis. Our study has however also some weaknesses: the choice to include only children between 2 and 5 years could lead to missing important factors associated with early life stunting. Further, information regarding the mother’s pregnancy, breastfeeding and early complementary feeding may be subject to recall bias. Also, due to the matched design, we could not assess for the effect of gender and we did not assess for birth spacing and paternal height, zinc or vitamin A status which are factors associated with stunting in previous studies.

In conclusion, our results suggest maternal education, antenatal care, iron supplementation and simple WASH interventions such access to soap and infection control and breastfeeding (Antananarivo) or better nutrition (Bangui) as specific interventions in the two study settings. Our results are an important milestone for these countries, as they provide a locally adapted roadmap for the implementation of targeted intervention and prevention strategies against childhood stunting.

## Supplementary Information

Below is the link to the electronic supplementary material.

Supplementary file1 (PDF 461 KB)
