# Supplementary material for: Factors Associated with Stunted Growth in Children Under Five Years in Antananarivo, Madagascar and Bangui, Central African Republic
Source: Matern Child Health J. 2021 Aug 12;25(10):1626–37. doi: 10.1007/s10995-021-03201-8 (PMC8448698; doi:10.1007/s10995-021-03201-8)
Supplement: Supplementary file 1 — Supplementary file1 (PDF 461 KB) [file 10995_2021_3201_MOESM1_ESM.pdf]

Factors associated with stunted growth in children under five years in Antananarivo, Madagascar and Bangui, Central African Republic

Pascale Vonaesch[1,2,3,#], Serge Ghislain Djorie[4,*], Kaleb Jephté Estimé Kandou[4,*], Maheninasy Rakotondrainipiana[5,*], Laura Schaeffer[6,*], Prisca Vega Andriatsalama[5], Ravaka Randriamparany[5], Bolmbaye Privat Gondje[7], Synthia Nigatoloum[7], Sonia Sandrine Vondo[7], Aurélie Etienne[5], Annick Robinson[8], Francis Allen Hunald[9], Lisette Raharimalala[10], Tamara Giles-Vernick[11], Laura Tondeur[6], Frédérique Randrianirina[12], Alexandra Bastaraud[13], Jean-Chrysostome Gody[7], Philippe Jean Sansonetti[1, 14$], Rindra Vatosoa Randremanana[5,$,#] for the AFRIBIOTA Investigators

1: Unité de Pathogénie Microbienne Moléculaire, Institut Pasteur, 25-28 Rue du Dr Roux, Paris
2: current address: Human and Animal Health Unit, Swiss Tropical and Public Health Institute, Socinstrasse 57, 4051 Basel, Switzerland
3: current address: University of Basel, 4051 Basel, Switzerland
4 : Unité d'Epidémiologie, Institut Pasteur de Bangui, Avenue de l'Indépendance, Bangui, Central African Republic
5: Unité Epidémiologie et de Recherche Clinique, Institut Pasteur de Madagascar, BP 1274, Antananarivo (101), Madagascar
6: Unité d'Epidémiologie des Maladies Emergentes, Institut Pasteur, 28 Rue du Dr. Roux, 75015 Paris, France
7: Centre Pédiatrique de Bangui, Avenue de l'Indépendance, Bangui, Central African Republic
8: Centre Hospitalier Universitaire Mère Enfant de Tsaralalana, rue Patrice Lumumba, Rue Mabizo S, Antananarivo (101), Madagascar
9: Service de Chirurgie pédiatrique, Centre Hospitalier Universitaire Joseph Ravoahangy Andrianavalona, BP 4150, Ampefiloha, Antananarivo (101), Madagascar
10: Centre de Santé Maternelle et Infantile de Tsaralalana, Lalana Andriantsilavo, Antananarivo (101), Madagascar
11: Anthropology and Ecology of Disease Emergence Unit, Institut Pasteur, 28 Rue du Dr. Roux, 75015 Paris, France
12: Centre de Biologie Clinique, Institut Pasteur de Madagascar, Madagascar, BP 1274, Antananarivo (101), Madagascar
13 : Laboratoire d'hygiène des aliments et de l'environnement (LHAE), Institut Pasteur de Madagascar, Madagascar, BP 1274, Antananarivo (101), Madagascar
14: current address: The Center for Microbes, Development and Health, Institut Pasteur of Shanghai and Chinese Academy of Sciences, 411 Hefei Rd, Huangpu, Shanghai, China

* these authors contributed equally to the work
[$] co-last authors
[#] co-corresponding authors

Factors associated with stunted growth in children under five years in Antananarivo, Madagascar and Bangui, Central African Republic, Vonaesch et al., Online Supplementary Material

Table S1: Characteristics of the mothers

	Bangui N=409	Antananarivo N=424	P-value
**Less than 15 years old at first**			Pr < 0.0001
**pregnancy**	71 (17.36%)	46 (11%)	
Yes	305 (75.5%)	373 (88%)	
No	33 (8%)	5 (1%)	
Unknown			
**Prenatal consultations(s)**			Pr = 0.002
Yes	390 (97%)	399 (94.5%)	
No	7 (2%)	23 (5.5%)	
Unknown	4 (1%)	0 (0%)	
**Place of giving birth**			Pr < 0.0001
Hospital	348 (85%)	164 (38.5%)	
Home	50 (12%)	248 (58.5%)	
Other	4 (1%)	10 (2.5%)	
Unknown	7 (2%)	2 (0.5%)	
**Nutritional status of mother***			Pr < 0.0001
Normal weight	234 (57%)	256 (60.5%)	
Underweight	56 (14%)	63 (14.5%)	
Overweight	66 (16%)	91 (21.5%)	
Unknown	53 (13%)	14 (3.5%)	
**Mothers height (cm)**	161.7 (161.0; 162.3)	152.5 (151.9; 153.0)	Pr < 0.0001
**Mothers education**			Pr < 0.0001
None	21 (5.5%)	13 (3%)	
Primary	151 (38%)	203 (48.5%)	
Middle school	168 (42.5%)	180 (43%)	
High school or more	55 (14%)	23 (5.5%)	
**Mother is working**			Pr < 0.0001
Yes	307 (75%)	266 (62.75%)	
No	93 (23%)	157 (37%)	
Unknown	9 (2%)	1 (0.25%)	

* based on the BMI of the mother; pregnant mothers were classified based on the Ververs criteria[1]: Mothers with amenorrhea for less than 13 weeks: undernourished if BMI <= 20.5; mothers with amenorrhea for 13 weeks to less than 27 weeks: undernourished if BMI <= 21.5; mothers with at least 27 weeks of amenorrhea: undernourished if BMI <= 22.5. Mothers were not classified if they were pregnant and had a BMI larger than 25 (missing value in this case as difficult to assess if this is a sign of overnutrition or not)

Table S2: Description of sanitary conditions

	Bangui N=409	Antananarivo N=424	P-value
**No times mother washes hand/day***	2.61 (2.53; 2.71)	4.16 (4.05; 2.53)	Pr < 0.0001
**Soap in household**			Pr < 0.0001
No or sometimes	344 (84%)	234 (55%)	
Always	65 (16%)	190 (45%)	
**Water for drinking is treated****	41 (10%)	114 (27%)	Pr < 0.0001
**Filtered drinking water**	3 (0.5%)	2 (0.5%)	Pr = 0.625
**Chlorinated drinking water**	36 (9%)	34 (8%)	Pr = 0.684
**Boiled drinking water**	2 (0.5%)	90 (21%)	Pr < 0.0001
**Water used in kitchen is treated**			Pr = 0.016
Yes	29 (7%)	51 (12%)	
No	380 (93%)	373 (88%)	
**Water for drinking/cooking is stored**			Pr = 0.005
Yes	6 (1.5%)	21 (5%)	
No	403(98.5%)	403 (95%)	
**Contamination source less 5m from house*****			Pr < 0.0001
Yes	140 (34%)	238 (56%)	
No	253 (62%)	83 (20%)	
Unknown	16 (4%)	103 (24%)	
**Disposal of wastewater**			Pr < 0.0001
In the street	213 (52%)	29 (7%)	
Open well in the house/garden	148 (36%)	19 (4.5%)	
Open well outside the house/garden	42 (10%)	321 (75.5%)	
Other	6 (2%)	55 (13%)	
**Disposal of waste**			Pr < 0.0001
Open well	93 (23%)	151 (35.5%)	
Closed well	16 (4%)	44 (10.5%)	
In the street	168 (41%)	57 (13.5%)	
Other	132 (32%)	172 (40.5%)	

* self-reported by mother, not counted
**water in the same household can be treated by several methods
*** self-reported

Table S3: Description of clinical features and comorbidities

	Bangui N=409	Antananarivo N=424	P-value
**Blood taken**			Pr < 0.0001
Yes	363 (89%)	413 (97.5%)	
No	46 (11%)	11 (2.5%)	
**Anemia (HB<11 g/l)***			Pr < 0.0001
Yes	167 (47%)	81 (20%)	
No	190 (53%)	332 (80%)	
**Ferritin low****			Pr < 0.0001
Normal (>=12 ng/ml)	333 (91%)	323 (78%)	
Low (< 12 ng/ml)	32 (9%)	89 (22%)	
**CRP level**			Pr < 0.0001
Normal (<= 10 mg/l)	284 (79%)	375 (91%)	
High (>10 mg/l)	74 (21%)	36 (9%)	
**Urinary iodine levels (µg/l)**	No data	101 (94.11; 107.88)	
**Vaccinations up to date**			Pr < 0.0001
Yes	2 (0.5%)	302 (71%)	
Incomplete	392 (96%)	117 (28%)	
No vaccinations	15 (3.5%)	3 (1%)	
**Dermatitis**			Pr < 0.0001
Yes	57 (14%)	27 (6%)	
No	352 (86%)	397 (94%)	
**Coughing**			Pr < 0.0001
Yes	211 (52%)	156 (37%)	
No	198 (48%)	268 (63%)	
**Clogged nose**			Pr < 0.0001
Yes	52 (13%)	222 (52%)	
No	357 (87%)	202 (48%)	
**Running nose**			Pr = 0.011
Yes	221 (54%)	266 (63%)	
No	188 (46%)	158 (37%)	
**Caries**			
Yes	Not	160 (38%)	
No	assessed	264 (62%)	
**Previous episode of severe acute undernutrition**			Pr < 0.0001
Yes	34 (8.25%)	10 (2%)	
No	374 (91.5%)	414 (98%)	
Unknown	1 (0.25%)	0 (0%)	
**Diarrhea at day of inclusion**			Pr = 0.001
Yes	0 (0%)	11 (2.5%)	
No	409 (100%)	413 (97.5%)	

* Hemoglobin levels for Madagascar are adapted for high altitude (WHO/NMH/NHD/MNM/11.1; http://www.who.int/vmnis/indicators/haemoglobin.pdf) ** Ferritin levels are adjusted for infection based on the CRP levels[2]

Table S4: Risk factors independently associated with stunting in the merged dataset*

	Non-stunted N= 426	Stunted N= 323	Unadjusted OR (95% CI)	Adjusted OR (95% CI)
**Mothers education**				
None	12 (3%)	20 (6%)	Ref	Ref
Primary	172 (40.5%)	160 (49.5%)	0.56 (0.26; 1.18)	0.76 (0.31; 1.84)
Middle school	181 (42.5%)	134 (1.5%)	0.44 (0.21; 0.94)	0.68 (0.28; 1.66)
High school or more	61 (14%)	9 (3%)	0.09 (0.03; 0.24)	0.15 (0.05; 0.45)
**Mother's height**				
More than 150 cm	343 (80.5%)	220 (68%)	Ref	Ref
Less than 150 cm	60 (14%)	83 (26%)	2.16 (1.49; 3.13)	2.52 (1.58; 4.03)
Unknown	23 (5.5%)	20 (6%)	1.36 (0.73; 2.53)	1.47 (0.40; 5.42)
**Nutritional status of mother***				
Normal weight	229 (54%)	189 (58.8%)	Ref	Ref
Underweight	48 (11%)	49 (15%)	1.24 (0.80; 1.92)	1.01 (0.60; 1.69)
Overweight	101 (24%)	45 (14%)	0.54 (0.36; 0.81)	0.46 (0.28; 0.73)
Unknown	48 (11%)	40 (12.5%)	1.01 (0.64; 1.60)	0.81 (0.40; 1.66)
**Age at first pregnancy**				
Less than 15 years	51 (12%)	58 (18%)	Ref	Ref
More than 15 years	367 (86%)	250 (77.5%)	0.60 (0.40; 0.90)	0.58 (0.36; 0.95)
Unknown	8 (2%)	15 (4.5%)	1.65 (0.65; 4.21)	5.80 (1.01; 33.47)
**Nutrition changed during pregnancy**				
No				
Yes	340 (80%)	277 (86%)	Ref	Ref
Unknown	72 (17%)	36 (11%)	0.61 (0.40; 0.94)	0.50 (0.30; 0.86)
	14 (3%)	10 (3%)	0.88 (0.38; 2.00)	0.11 (0.02; 0.86)

**Partially breastfed ≥ 12 months**				
**No**	25 (6%)	39 (12%)	Ref	Ref
**Yes**	380 (89%)	268 (83%)	[illegible].45 (0.27; 0.76)	0.45 (0.24; 0.85)
**Unknown**	21 (5%)	16 (5%)	[illegible]49 (0.21; 1.11)	0.30 (0.11; 0.82)
**Soap available in household**				
Yes	319 (75%)	178 (55%)	Ref	Ref
No or only sometimes	107 (25%)	145 (45%)	2.43 (1.78; 3.31)	1.97 (1.35; 2.89)
**Anemia (Hb <11 g/l)**				
No	324 (76%)	187 (58%)	Ref	Ref
Yes	102 (24%)	136 (42%)	2.31 (1.69; 3.16)	2.83 (1.89; 4.22)
**Birth weight ****				
Average	162 (38%)	139 (43%)	Ref	Ref
Smaller than average	46 (11%)	83 (25.5%)	2.10 (1.37; 3.22)	2.32 (1.40; 3.85)
Bigger than average	144 (34%)	64 (20%)	0.52 (0.36; 0.75)	0.46 (0.29; 0.72)
Unknown	74 (17%)	37 (11.5%)	0.58 (0.37; 0.92)	0.58 (0.33; 1.04)
**Diarrhea at time of inclusion**				
No	425 (99.75%)	313 (97%)	Ref	Ref
Yes	1 (0.25%)	10 (3%)	13.58 (1.73; 106.62)	12.13 (1.38; 106.38)
**Dermatitis at time of inclusion**				
No	393 (92%)	282 (87%)	Ref	Ref
Yes	33 (8%)	41 (13%)	1.73 (1.07; 2.81)	1.92 (1.09; 3.38)
**Previous episode of severe acute undernutrition**				
No	421 (99%)	292 (90%)	Ref	Ref
Yes	5 (1%)	31 (10%)	8.94 (3.44; 23.26)	5.96 (2.09; 17.04)

* Analysis performed on 749 subjects; ** as perceived and remembered by mothers/ main caregivers; $adjusted for season of inclusion, age in years, gender and country of inclusion (matching) as well as the other independently associated variables.

Extended Material and Methods

Study design and study population

The AFRIBIOTA study[3] is a case-control study for stunting in children aged 2-5 years in Bangui, CAR and Antananarivo, Madagascar. Inclusion criteria were HIV-negative children, neither suffering from acute malnutrition nor from any other severe disease, living in the 6st, 7st or 8th district of Bangui, CAR or in two neighbourhoods of Antananarivo, Madagascar (Ankasina or

Andranomanalina Isotry). Children were recruited in the community by health workers and screened for eligibility during inclusion sessions at the local health district. Recruitment took place within these districts or neighbourhoods, and included children were admitted to the hospital for sample collection (blood, stool, duodenal and gastric aspirates) and anthropometric measurement. Sample tacking and interviewing took place centrally at the hospital. Stunting severity was defined based on the WHO 2006 standards, as described below. Stunted and control children were matched according to age (24-35 months, 36-47 months and 48-60 months), gender, neighbourhood and season of inclusion (dry or wet season). Recruitment took place between December 2016 and March 2018 in Antananarivo and between January 2017 and May 2018 in Bangui.

Sample size estimation
Sample size for AFRIBIOTA was calculated to respond to the primary objective, which was to find differences in the microbiota of stunted compared to non-stunted children. Analysis of factors associated with stunting was one of the study's secondary objectives. Assuming an alpha-error of 0.05%, a power of 80% and an expected 20% exposure in the cases, we needed at least 169 children per group. The initial total targeted sample size was 920 children. In each country, we intended to enrol stunted children (200/country) and children with no growth delay (260/country). The final sample size analysed in this study was of 836 children (Fig. 1).

Anthropometric measurements
Anthropometric variables included height, weight, head circumference and mid-upper arm circumference (MUAC). Height was measured on standing children, and weight with minimal clothing (underwear). All anthropometric measurements were taken twice and repeated, if the height or MUAC differed by more than 1 mm or the weight by more than 100g between the two measurements. The following variables were calculated using the WHO Anthro software (version 3.2.2, January 2011): height-for-age z-score (HAZ), weight-for-age z-score (WAZ), BMI-for-age z-score (zBMI), weight-for-height z-score. We used the following cut-offs defined by the WHO Growth Child standards 2006[4,5] : stunted: < -2 HAZ (moderately stunted: -3 ≤ HAZ < -2; severely stunted: HAZ < -3); acute malnutrition based on WHZ score: < -2 WHZ acutely malnourished).

Data collection
A standardized, paper questionnaire in French that was translated *ad hoc* to the local languages Sangho and Malagasy was used in both study sites and data was entered in double in an Access database. The questionnaire was given by trained data clerk (Bangui) or trained medical doctors (Antananarivo). Trained medical doctors performed all clinical examinations. The CRF included information about children's age, gender, family structure, socioeconomic status indicators (e.g. profession of parents, working

situation, household size, description of living conditions, household assets, means of transportation), sanitary indicators (e.g. drinking water, eating with fingers, access to sanitary facilities), data about the mother's pregnancy and child's and family nutrition and feeding practices (e.g. 24 hour recall, number of meals, breastfeeding). Each child was also examined by a medical doctor and comorbidities such as caries, non-severe respiratory complaints, mild diarrhoea and/or dermatitis were assessed. For each child, venous blood was further collected. Complete blood count, C-reactive protein (CRP) and ferritin levels were measured at the Institut Pasteur de Madagascar and the Institut Pasteur de Bangui within 4 hours after blood collection according to accredited methods. Ferritin levels were corrected for systemic inflammation[2] haemoglobin values were adjusted for altitude[6,7] and anaemia was defined as less than 110 g/l, according to WHO criteria[5,8]. A dietary diversity score (DDS) was calculated based on a 24 hour recall based on the seven good groups defined by WHO: (1) grains, roots and tubers; (2) legumes and nuts; (3) dairy products; (4) flesh foods (meats/fish/poultry); (5) eggs; (6) vitamin A-rich fruits and vegetables; and (7) other fruits and vegetables. According to WHO standards, a DDS of four is considered the minimum DDS for normal food diversity[9]. Mother's nutritional status was based on the Body Mass Index (BMI; weight (kilogram) divided by the square of body height (meter)). Non-pregnant mothers were classified by BMI categories as underweight if BMI was <18.5 kg/m², normal weight if BMI was ≥ 18.5kg/m² and ≤ 25kg/m², and overweight if BMI was >25kg/m². Pregnant mothers were classified according to the categories proposed by Ververs[1] : BMI <20.5 kg/m² if amenorrhea less than 13 weeks, BMI <21.5 kg/m² if amenorrhea between 13-27 weeks, and BMI <22.5 kg/m² if amenorrhea is 27 weeks or more. A wealth index was created based on the minimal set of assets, leading to a separation of subjects in three distinct groups in a principal component analysis (PCoA). The minimal set of assets included housing materials (floor and wall materials, ownership of a car, telephone, bike, motorcycle), access to specific utilities (electricity, bathroom, cooking location), and family size. Iteration tests were then performed to check whether the selected variables reflect the observed clusters. We defined three household wealth categories according to the cluster observed: poorest, middle and wealthiest category. The categories were defined as follows: lowest socioeconomic score: no telephone or house floor made of pounded earth; middle socioeconomic score: telephone, wooden or concrete house floor, no internal shower or internal kitchen; highest score: telephone, wooden or concrete house floor, either an internal shower or internal kitchen (separate room).

Statistical analysis

The statistical analysis was performed with Stata 13. Significance level was fixed for all analyses at 0.05 and all tests were performed bilaterally. Categorical variables were expressed as percentages; quantitative variables were expressed as a mean (± Standard Deviation) or median (interquartile range). The stunted vs. non-stunted groups were compared using Chi2 or Fisher Exact test for qualitative variables and the Student t test or the Mann-Whitney U test for quantitative variables. All variables were assessed in a bivariate analysis. Factors associated with stunting in bivariate analysis with a p-value of <0.2 were checked for

potential confounding factors and interactions and then included in a backward logistic regression. As there was not a perfect matching for age, gender and season of inclusion, these variables were forced in the multivariate model. Results are reported as adjusted OR with 95% CI, corrected for age in years, gender, season of inclusion and country of origin.

Ethical considerations

The AFRIBIOTA study protocol was approved by the Institutional Review Board of the Institut Pasteur (2016-06/IRB) and the national ethical review boards of Madagascar (55/MSANP/CE) and the Central African Republic (173/UB/FACSS/CSCVPER/16). Legal representatives of all child participants received oral and written information about the study and provided written consent for their children to participate in the study.

Extended Acknowledgements

The authors wish to thank all participating families, the AFRIBIOTA Consortium, including all field workers, laboratory engineers, technicians, administrative support persons, doctors and nurses, the participating hospitals in Bangui and Antananarivo (Complexe Pédiatrique de Bangui, Centre Hospitalier Universitaire Mère-Enfant de Tsaralalàna (CHUMET), Service de Chirurgie pédiatrique du Centre Hospitalier Universitaire Joseph Ravoahangy Andrianavalona (CHUJRA) and Centre de Santé Maternelle et Infantile de Tsaralalàna), the Office National de Nutrition de Madagascar and the Office Régional de Nutrition Analamanga, the Direction de Lutte contre les Infections Sexuellement Transmissibles de Madagascar, the nutrition section of UNICEF in Bangui, Central African Republic, the local health centers in Bangui (St. Joseph de Galabadja, Centre de Santé de Pétévo and Centre de Santé de St. Paul) and in Antananarivo (Sites de nutrition communautaire Ankasina et Andranomanalina Isotry) as well as the community health workers and administrative authorities in the corresponding arrondissements and quartiers. We also wish to thank the Institut Pasteur, the Institut Pasteur de Madagascar and the Institut Pasteur de Bangui for their continuous support. The authors wish to thank the Centre de Recherche Translationelle and the Direction Internationale of the Institut Pasteur, especially Paméla Palvadeau, Jane Lynda Deuve, Cécile Artaud, Nathalie Jolly for precious help in setting-up and steering the AFRIBIOTA project. We further thank Lisa Crump and Astrid Knoblauch for critical reading of the manuscript.

References

1 Ververs, M. T., Antierens, A., Sackl, A., Staderini, N. & Captier, V. Which anthropometric indicators identify a pregnant woman as acutely malnourished and predict adverse birth outcomes in the humanitarian context? *PLoS Curr* **5**, doi:10.1371/currents.dis.54a8b618c1bc031ea140e3f2934599c8 (2013).

2 Thurnham, D. I. *et al.* Adjusting plasma ferritin concentrations to remove the effects of subclinical inflammation in the assessment of iron deficiency: a meta-analysis. *Am J Clin Nutr* **92**, 546-555, doi:10.3945/ajcn.2010.29284 (2010).

3 Vonaesch, P. *et al.* Identifying the etiology and pathophysiology underlying stunting and environmental enteropathy: study protocol of the AFRIBIOTA project. *BMC pediatrics* **18**, 236, doi:10.1186/s12887-018-1189-5 (2018).

4 Group, W. M. G. R. S. *… Standards: Head circumference-for-age, arm circumference-for-age, triceps skinfold-for-age and subscapular skinfold-for-age: Methods and development*. (Geneva: World Health …, 2007).

5 Onis, M. WHO Child Growth Standards based on length/height, weight and age. *Acta paediatrica* **95**, 76-85, doi:10.1111/j.1651-2227.2006.tb02378.x (2006).

6 Centers for Disease, C. CDC criteria for anemia in children and childbearing-aged women. *MMWR Morb Mortal Wkly Rep* **38**, 400-404 (1989).

7 Sullivan, K. M., Mei, Z., Grummer-Strawn, L. & Parvanta, I. Haemoglobin adjustments to define anaemia. *Trop Med Int Health* **13**, 1267-1271, doi:10.1111/j.1365-3156.2008.02143.x (2008).

8 OMS. *Concentrations en hémoglobine permettant de diagnostiquer l'anémie et d'en évaluer la sévérité*, 2011).

9 World Health Organization. Indicators for assessing infant and young child feeding practices: conclusions of a consensus meeting held. (2007).
